# Central COVID-19 Coordination Centers in Germany: Description, Economic Evaluation, and Systematic Review

**DOI:** 10.2196/33509

**Published:** 2021-11-18

**Authors:** Nikolas Schopow, Georg Osterhoff, Nikolaus von Dercks, Felix Girrbach, Christoph Josten, Sebastian Stehr, Pierre Hepp

**Affiliations:** 1 Department for Orthopedics, Trauma Surgery and Plastic Surgery University Hospital Leipzig Leipzig Germany; 2 Medical Controlling University Hospital Leipzig Leipzig Germany; 3 Department of Anesthesiology and Intensive Care Medicine University Hospital Leipzig Leipzig Germany

**Keywords:** telemedical consultation, patient allocation, algorithm-based treatment, telemedicine, telehealth, consultation, allocation, algorithm, treatment, COVID-19, coordination, Germany, economic, review, establishment, management

## Abstract

**Background:**

During the COVID-19 pandemic, Central COVID-19 Coordination Centers (CCCCs) have been established at several hospitals across Germany with the intention to assist local health care professionals in efficiently referring patients with suspected or confirmed SARS-CoV-2 infection to regional hospitals and therefore to prevent the collapse of local health system structures. In addition, these centers coordinate interhospital transfers of patients with COVID-19 and provide or arrange specialized telemedical consultations.

**Objective:**

This study describes the establishment and management of a CCCC at a German university hospital.

**Methods:**

We performed economic analyses (cost, cost-effectiveness, use, and utility) according to the CHEERS (Consolidated Health Economic Evaluation Reporting Standards) criteria. Additionally, we conducted a systematic review to identify publications on similar institutions worldwide. The 2 months with the highest local incidence of COVID-19 cases (December 2020 and January 2021) were considered.

**Results:**

During this time, 17.3 requests per day were made to the CCCC regarding admission or transfer of patients with COVID-19. The majority of requests were made by emergency medical services (601/1068, 56.3%), patients with an average age of 71.8 (SD 17.2) years were involved, and for 737 of 1068 cases (69%), SARS-CoV-2 had already been detected by a positive polymerase chain reaction test. In 59.8% (639/1068) of the concerned patients, further treatment by a general practitioner or outpatient presentation in a hospital could be initiated after appropriate advice, 27.2% (291/1068) of patients were admitted to normal wards, and 12.9% (138/1068) were directly transmitted to an intensive care unit. The operating costs of the CCCC amounted to more than €52,000 (US $60,031) per month. Of the 334 patients with detected SARS-CoV-2 who were referred via EMS or outpatient physicians, 302 (90.4%) were triaged and announced in advance by the CCCC. No other published economic analysis of COVID-19 coordination or management institutions at hospitals could be found.

**Conclusions:**

Despite the high cost of the CCCC, we were able to show that it is a beneficial concept to both the providing hospital and the public health system. However, the most important benefits of the CCCC are that it prevents hospitals from being overrun by patients and that it avoids situations in which physicians must weigh one patient’s life against another’s.

## Introduction

COVID-19 has infected more than 230 million people, including over 4 million people in Germany (as of September 2021) [1], since it was declared a global pandemic by the World Health Organization on March 11, 2020 [2]. Due to large numbers of hospital admissions of patients with COVID-19 within a very short time, catastrophic overloads of hospitals have repeatedly occurred worldwide, as observed in Bergamo [3] and New York City [4].

In the event that intensive care units (ICUs) are overcrowded, patients must be transferred to more distant hospitals by intensive care transport. However, interhospital transport of critically ill patients always involves a high risk for the patient (eg, dislocation of intravascular catheters or airway devices) and should therefore be avoided if possible.

Emergency medical services (EMS) in Germany are usually dispatched by a regional rescue directing center, where emergency calls are handled by specially trained firefighters or paramedics. During the pandemic, however, a special coordination center with an up-to-date overview of the highly dynamic capacities of the surrounding hospitals became necessary. Main tasks have included triage of suspected and confirmed patients with COVID-19, coordination of secondary patient transfers of critically ill patients requiring intensive care based on current hospital capacity, and the arrangement of specialist telemedical consultations for peripheral hospitals in need of expertise in the treatment of patients with COVID-19. The staff deployed thus need to be able to use the information given via telephone to advise outpatients on further medical care and, if an inpatient admission is necessary, to estimate the correct level of care now and in advance at the hospital. This would significantly exceed the capacities of the rescue control center, which is why the CCCCs as separate coordination centers with permanent medical staffing were introduced.

The main goals of the CCCCs were to implement an efficient distribution of patients with COVID-19 to provide the best medical care to all and to reduce interhospital transfers of patients with COVID-19 to a minimum.

Therefore, on behalf of the state government, three CCCCs were established in Saxony, Germany, located at Dresden University Hospital for eastern Saxony, Chemnitz Hospital for southwestern Saxony, and Leipzig University Hospital (LUH) for northern Saxony. The CCCC at LUH is responsible for the coordination of 18 hospitals, 112 EMS vehicles, and over 700 primary care physicians [5].

The following article aims to describe the structure of the CCCC at LUH and to perform an economic evaluation of the two months with the highest incidence in the second COVID-19 wave (December 2020 to January 2021, local incidences >500/100,000/week) [6]. In addition, we conducted a systematic literature review on economic data for similar coordination units.

## Methods

The study was approved by the Local Ethics Committee (158/21-ek). The literature review was conducted according to the PRISMA (Preferred Reporting Items for Systematic Reviews and Meta-Analyses) 2020 guidelines [7], and the economical evaluation was performed according to the CHEERS (Consolidated Health Economic Evaluation Reporting Standards) guidelines [8].

### Systematic Review

A search of published records was conducted using the following equations: (COVID* OR SARS*) AND (Coordination* OR Management*) AND Cost*, in the PubMed (n=555) and Web of Science (n=767) databases (last update 07/15/2021). The search was not restricted to any field. First, all publications before 2020 (n=144) were removed, followed by all duplicates (n=295). For this review, full-text availability articles published in peer-reviewed journals and written in English or German were considered. Abstracts and conference proceedings were excluded (n=109). In addition, we investigated the reference lists of the articles. The articles were required to meet the quality standards of CHEERS.

### Setup of the Central COVID-19 Coordination Center

**Figure 1:**
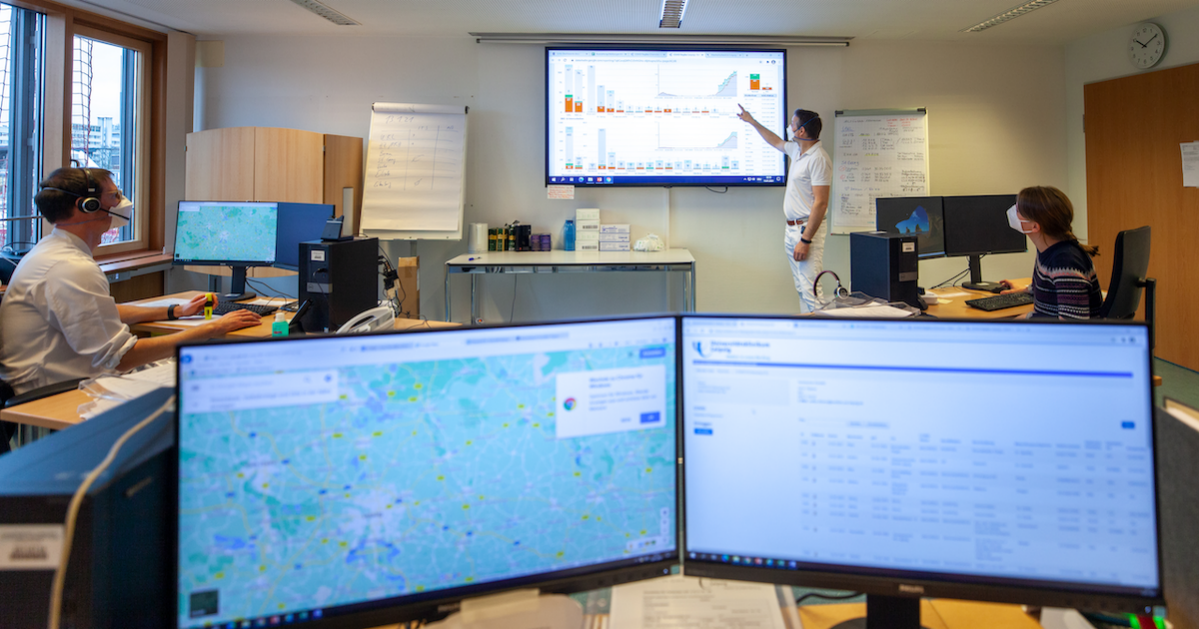
Setup of the Central COVID-19 Coordination Center at Leipzig University Hospital, including a conference room (51 square meters), a central widescreen display (dashboard), three computer workstations with telephones, two whiteboards, and a multifunction printer (not shown). The center is staffed in the early shift by two physicians (right and left), and the Deputy Chief of hospital emergency management is shown in front of the dashboard (center).

The CCCC at LUH is staffed 24 hours per day, 7 days per week in a 4-shift system by physicians (early duty, 2 physicians; mid-duty, 1 physician; late duty, 1 physician; and night duty, 1 physician). Medical students and nurses are also assigned to the overlapping mid-shift duty. A 51 m^2^ conference room equipped with three workstations was chosen (Figure 1).

**Figure 2:**
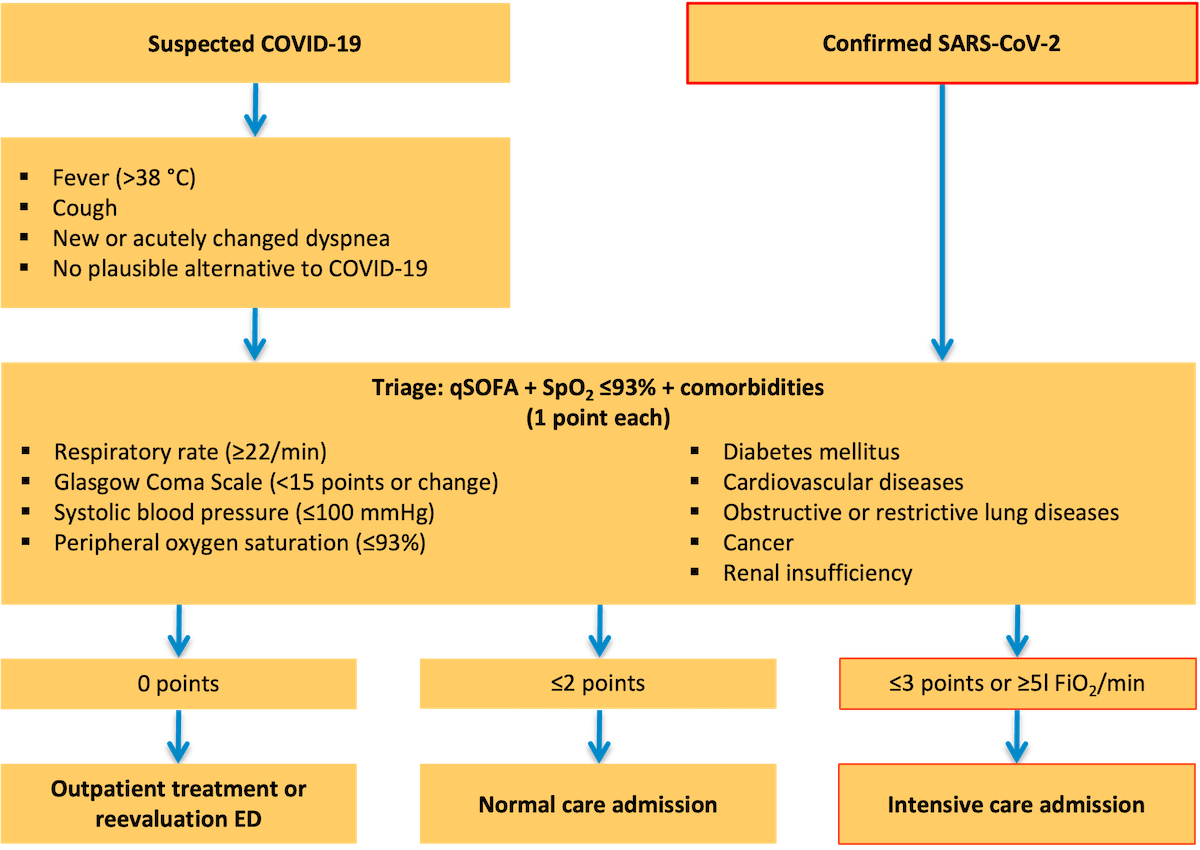
Algorithm of the Central COVID-19 Coordination Center at Leipzig University Hospital for handling requests from emergency medical services or outpatient physicians for suspected COVID-19 or SARS-CoV-2 confirmation. Based on the algorithm of Central COVID-19 Coordination Center Dresden (simplified presentation). ED: emergency department; FiO_2_: fraction of inspired oxygen; qSOFA: quick sepsis-related organ failure assessment score; SpO_2_: peripheral oxygen saturation.

When requests were received, patient history and triage were performed according to a predetermined algorithm (Figure 2). The allocation was made based on the current bed capacity, which was displayed on a specially developed dashboard, and after telephone consultation with the target hospital. The queried information and the derived decision were documented in a database.

Either the specialized telemedical consultation was performed by the CCCC staff themselves, or the request was forwarded to appropriate specialists of the LUH (eg, inquiries regarding extracorporeal membrane oxygenation treatment due to severe lung failure, hemostasiological issues).

### Requests and Patients

All received requests at the CCCC at LUH in December 2020 and January 2021 were included and analyzed in this evaluation. The time of each request, information about the requesters (contact person, function), the epidemiological data of the patients, and derived decisions were documented in a specially developed database and analyzed for this study.

### Cost Analysis

**Figure 3:**
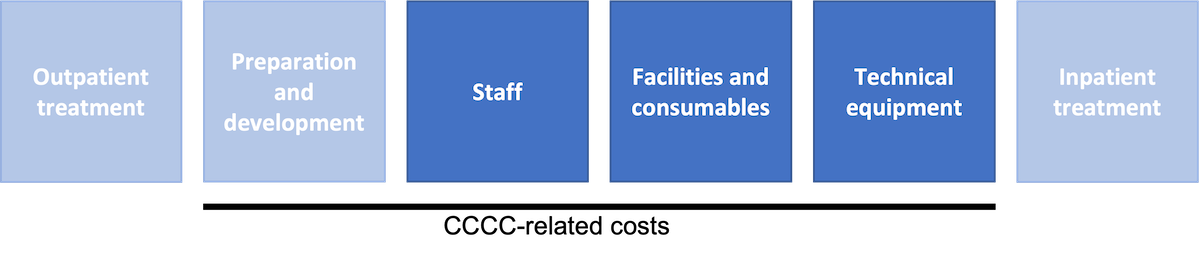
Calculation model of the costs of the Central COVID-19 Coordination Center at Leipzig University Hospital.

To calculate the total ongoing costs of the CCCC at LUH, we chose a modular model (Figure 3). We did not consider the organizational costs previously incurred at LUH; development costs of the dashboard, database, and associated forms; out- and inpatient care costs; or construction-related costs (these were omitted due to dual hospital financing in the German health care system from the hospitals’ perspective). We also did not consider indirect or intangible costs (eg, loss of personnel and resulting reduction in treatment capacity in the providing hospitals).

#### Staff

The staff costs correspond to the payroll of the human resources department for December 2020 and January 2021. The costs are listed separately according to grade (physicians, nurses, and medical students) and include all ancillary staff costs as well as working hours and holiday bonuses. Also considered separately are costs of the CCCC front-office services, back-office services (telemedicine consulting), and administrative activities (management and scheduling).

#### Facilities and Consumables

The selected conference room had a size of 51 m^2^. The costs consist of operating costs (cleaning, energy, etc), consumables, and rent. The consumables (printer paper, whiteboard, etc) were calculated at a flat rate of 10€ (US $11.54) per day. The furnishings (chairs, desks, etc) were borrowed from the existing inventory; thus, no costs were incurred. The costs recorded correspond to the costs in 2020. For the calculation of the costs in 2021, they were increased by 4.1%, in accordance with the average development of material costs in German hospitals [9].

#### Technical Equipment

The technical equipment of the CCCC at LUH was newly purchased; the equipment will be used further after the end of the pandemic, and the costs will be depreciated over 4 years. We assume that the CCCC setup will exist for a total of 24 months, although not continuously in active operation; therefore, the running costs in each of the 2 months correspond to 1/12 of the annual depreciation. The cost of the multifunction printer is a blended monthly bill of lease, rental, and cost per printed page.

### Cost-effectiveness Analysis

The internal economic evaluation of the CCCC includes a comparison of costs, requests, and workload. For this purpose, separate documentation was conducted by the CCCC front office staff in April 2021 (with a similar number of requests as in December 2020 and January 2021). In this process, the length of time spent processing requests was recorded (from the ringing of the telephone until completion of documentation) as well as the amount of work spent on other tasks. The results were compared with the employees’ working hours.

### Use and Utility Analysis

This investigation examined how many patients with SARS-CoV-2 infection were treated in the ED of the LUH (self-, EMS-, or physician-initiated presentations) or admitted via this department (transfers from other hospitals). For this purpose, an evaluation was performed in the investigated period and in an analogous period from December 2019 to January 2020 via the hospital information system. The results were compared with the decisions of the CCCC. In addition, the attending physicians of the ED were interviewed.

## Results

### Systematic Review

**Figure 4:**
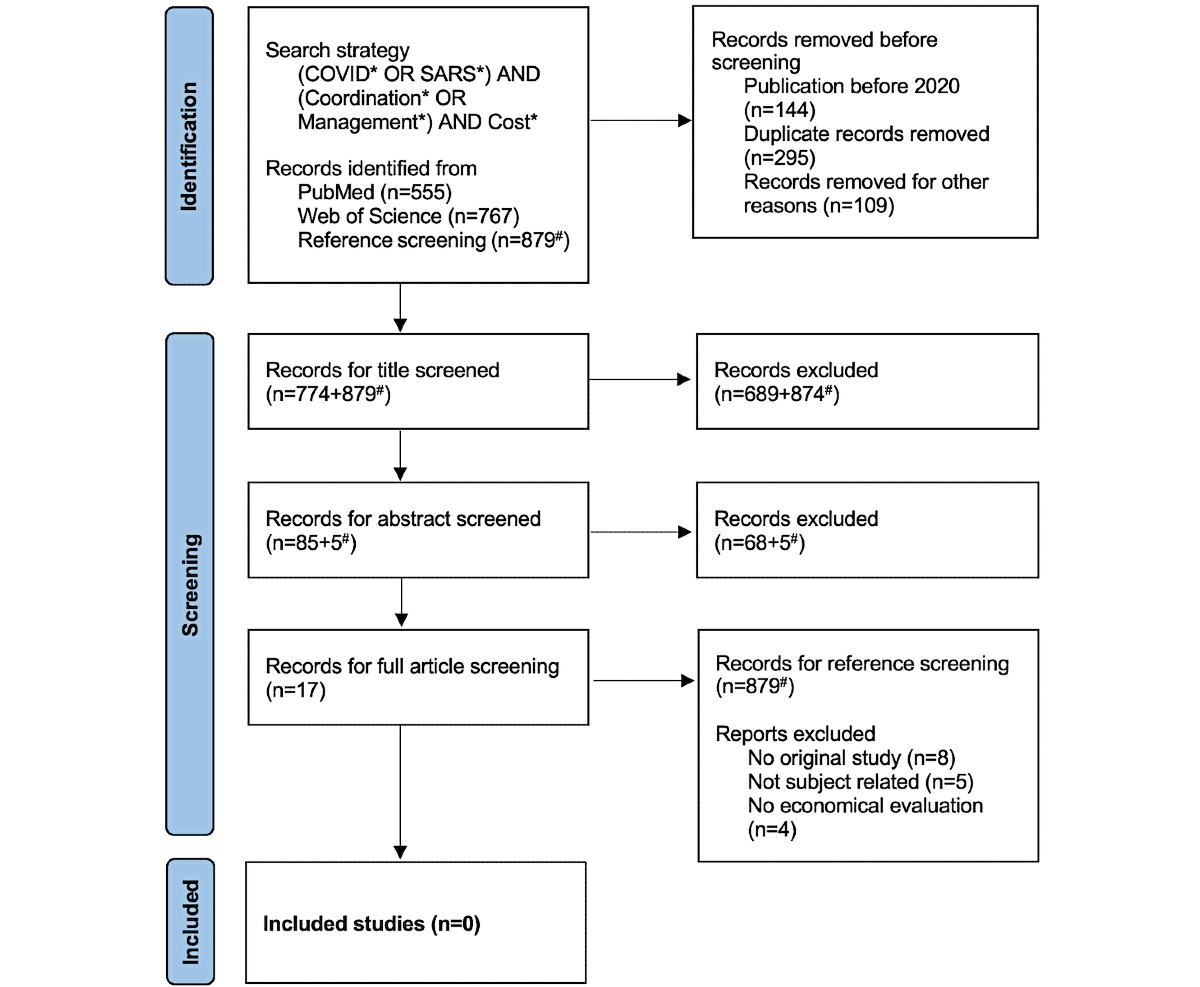
PRISMA (Preferred Reporting Items for Systematic Reviews and Meta-Analyses) flow diagram of the systematic review. Last update July 15, 2021. Numbers marked with # are based on the reference screening and are not included in the records removed before screening.

The PRISMA flow diagram for the literature analysis is shown in Figure 4. A total of 2201 publications were reviewed. No studies were found that addressed the costs of coordination or management tasks in the COVID-19 pandemic in regional or national health care systems. Moreover, the reference lists of full-text screened articles were screened and did not reveal any relevant publication (marked by # in Figure 4).

### Requests and Patients

**Figure 5:**
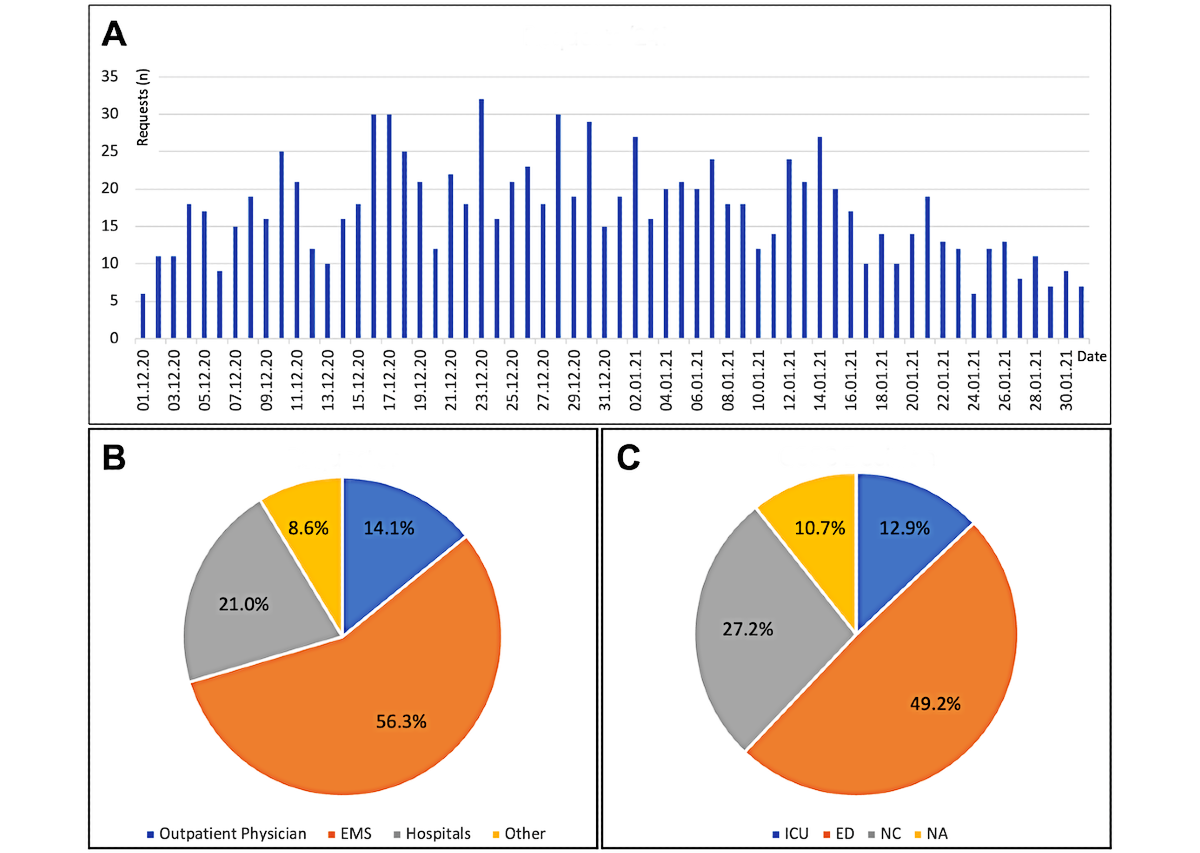
Requests and decisions of the Central COVID-19 Coordination Center (CCCC) at Leipzig University Hospital between December 01, 2020, and January 31, 2021. (A) Quantities of requests per 24 hours. (B) Proportions of different requestors. (C) Decisions by the CCCC after questions and consultation. ED: emergency department; EMS: emergency medical service; ICU: intensive care unit; NA: no admission; NC: normal care unit.

Between December 01, 2020, and January 31, 2021, 1068 telephone inquiries were accepted by the CCCC at LUH (Figure 5), with a mean value of 18.9 requests (SD 6.7) per day in December 2020, and 15.6 requests per day (SD 5.8) in January 2021 (Figure 5A). In the period under investigation, 56.3% (601/1068) of the requests were made by the EMS, 21.0% (224/1068) by hospitals, 14.1% (151/1068) by outpatient physicians (general practitioners), and 8.6% (92/1068) by others (Figure 5B).

Requests were made for patients aged 0 to 100 years, with an average age of 72 years, and 69% of cases that presented with SARS-CoV-2 infection (737/1068) were confirmed by polymerase chain reaction at the time of inquiry. Approximately one-fifth of the patients (200/1068, 18.7%) were suspected or detected SARS-CoV-2 positive by rapid test, and 12.3% (131/1068) had no detection or suspicion (Table 1).

At the time of the request, 97 of the 1068 patients (9.1%) had a respiratory rate >22/min, 576 (53.9%) showed peripheral oxygen saturation ≤93%, and 30 (2.8%) presented a systolic blood pressure <100 mmHg. Outpatient treatment or telephone consultation was sufficient for 59.8% (639/1068) of all requests, and inpatient treatment was needed for 40.2% (429/1068) of requests (Figure 5C).

**Table 1:** Patient data of requests to the Central COVID-19 Coordination Center at the Leipzig University Hospital between December 01, 2020, and January 31, 2021 (N=1068).

Characteristic		Value
**Epidemiological data**		
	Female, n (%)	475 (44.5)
	Mean age, mean (SD)	71.8 (17.2)
**SARS-CoV-2 status, n (%)**		
	Polymerase chain reaction test positive	737 (69)
	Suspicion/rapid test positive	200 (18.7)
	No suspicion	131 (12.3)
**Previous diseases, n (%)**		
	Diabetes mellitus	142 (13.3)
	Cardiovascular diseases	292 (27.3)
	COPD^a^/bronchial asthma	106 (9.9)
	Malignant neoplasia	47 (4.4)
	Renal insufficiency	109 (10.2)
	No relevant comorbidity	551 (51.6)
**Current symptoms, n (%)**		
	Respiratory rate (≥22/min)	97 (9.1)
	GCS^b^ (<15 or change) staff cost (€)^c^	33 (3.1)
	Systolic blood pressure (≤100 mmHg)	30 (2.8)
	SpO_2_^d^ (≤93%)	576 (53.9)
**Points in triage, n (%)**		
	0 points	281 (26.3)
	1-2 points	624 (58.4)
	≥3 points	163 (15.3)

^a^COPD: chronic obstructive pulmonary disease.

^b^GCS: Glasgow Coma Scale.

^c^1€=US $1.15.

^d^SpO_2_: peripheral oxygen saturation.

### Cost Analysis

Detailed costs and total costs are presented in Table 2, separately for December 2020 and January 2021 and in total.

**Table 2:** Detailed cost report of the Central COVID-19 Coordination Center at the Leipzig University Hospital between December 01, 2020, and January 31, 2021.

Characteristic			Staff cost (€)^a^				
			12/2020		01/2021		Total
**Front office**			41,067.99		43,849.02		84,917.01
	Physician	34,816.57		40,294.38		75,110.95	
	Nurse	5998.61		3554.64		9553.25	
	Student assistant	252.81		N/A^b^		252.81	
Back office			6355.26		N/A		6355.26
Administration			6207.12		6207.12		12,414.24
**Facilities and consumables**							
	Rent	305.64		318.17		623.81	
	Operating costs	472.72		492.10		964.83	
	Consumables	310.00		322.71		632.71	
**Technical equipment**							
	Wide screen display (n=1)	666.67		694.00		1360.67	
	Computers (n=4)	243.77		253.76		497.53	
	Monitors (n=6)	66.15		68.86		135.01	
	Desktop telephones (n=3)	50.00		52.05		102.05	
	DECT^c^ telephones (n=3)	75.0		78.08		153.08	
	Multifunction printer (n=1)	74.41		64.04		138.45	
Total			55,894.73		52,399.92		108,294.65

^a^1€=US $1.15.

^b^N/A: not applicable.

^c^DECT: digital enhanced cordless telecommunications.

### Cost-effectiveness Analysis

During 10 shifts in early and late duty, 74 calls were documented. Out of these, 23 calls were of informative or consulting character, and 51 concerned admission or transfer of patients. The average duration of work per request was 15.7 minutes (range 2-110 minutes, consultation: 10.2 minutes, admission: 18.1 minutes). This resulted in a workload of 24.1% of the working time at the front office.

### Use and Utility Analysis

At LUH, 4873 patients were treated or admitted via the ED during the investigated period. A total of 736 of these 4873 patients required isolation (15.1%, compared to 9.5% [577/6049] from December 2019 to January 2020); 7.2% (352/4873) because of SARS-CoV-2 (compared to 0% [0/6049]), 6.5% (318/4873) because of multidrug-resistant bacteria (compared to 8.2% [493/6049]), and 1.4% (66/4873) for other causes, such as immune-suppressed or other viral diseases (compared to 1.4% [84/6049]).

SARS-CoV-2 was detected in 352 patients, of whom 334 (94.8%) were referred via EMS or outpatient physicians. Among these 334 patients, 302 admissions or transfers were referred to LUH by the CCCC during the same period (90.4%). During the whole period that the CCCC was in operation, the ED was never overcrowded with patients with COVID-19.

## Discussion

### Principal Findings

For regional management of prehospital and in-hospital patients with COVID-19, a supportive unit was created at a tertiary hospital in Germany. The use and utility analysis underlines the benefit of the CCCC, whereas the health economic analysis shows potential for improvement in cost-effectiveness. In the additionally conducted systematic review, no studies of similar units could be found. Analyses addressing the coordination of other pandemics were also not found, although the establishment of similar regional, national, and international facilities was repeatedly requested in relevant literature [10-12].

Public health studies on coordination units for managing mass casualty incidents caused by accidents or natural disasters have been performed. So-called Disaster Medical Assistant Teams are used in various countries and can also support the logistical organization, but economic analyses are lacking [13-16].

Successful telemedical approaches already exist in the preclinical care of severely injured people [17,18]. A national program for telemedicine consultation after neurotrauma could eliminate the need for 68% of patient transfers [19].

A reduction in mortality of patients requiring intensive care after telemedicine consultation was also recently shown in a meta-analysis of 13 studies [20]. The successful implementation in other countries of supportive coordination units in disaster medicine and the good results of prehospital telemedicine consultation in the ED and ICU underline the joint approach of CCCCs in Germany.

Dealing with disasters and pandemics requires collaboration, coordination, and management [21,22]. Before the SARS-CoV-2 pandemic, major viral outbreaks such as severe acute respiratory syndrome in 2002, H1N1 in 2009, Middle East respiratory syndrome in 2012, H7N9 in 2013, Ebola virus in 2014, Zika virus in 2015, and dengue virus in 2020 have demonstrated that emergency management is essential to minimize damage to populations and economies [23-29]. Even developed countries with otherwise highly functional health care systems, such as the United States, United Kingdom, and Italy, observed a (time-limited) regional collapse of their health care systems.

To prevent similar situations in Germany, CCCCs have been set up in several regions across the country. These centers provide advice and support for the admission and transfer of patients. Here, we describe the structure, economic considerations, and benefits of a coordination center in one of the most severely affected regions of Germany.

In our systematic review, we could not find any similar previous studies on this topic.

By centralizing coordination, it was possible to establish a standardized procedure very early and thus make transparent decisions for all coordinated hospitals, which supported outpatient physicians and the EMS. At the beginning of the pandemic, when the CCCCs were formed, established decision-supporting algorithms were only available for other diseases. Therefore, the algorithm (Figure 2) is based on a combination of the quick sepsis-related organ failure assessment (qSOFA) score, which was actually developed for early sepsis detection [30] (the normal SOFA score does not seem to be optimal for risk stratification in patients with COVID-19, so adjustments may be necessary [31]); oxygen saturation to estimate oxygenation disturbance; and pre-existing conditions that predispose patients to severe COVID-19 progression [32].

To increase the acceptance of the CCCCs’ recommendations, we decided that they should be staffed primarily by physicians. The cost analysis shows that the majority of the costs of the CCCCs are contributed by human resources. Administrative activities (mainly planning and organization) and back-office activities (specialty physician consultation) together represent less than 20% of the total costs. We do not see any possibility of saving administrative costs due to the dynamic situation and constantly necessary adjustments. The costs for specialist consulting could decrease in the future as the experience of external colleagues increases and the requests become fewer. The front office personnel costs are responsible for 78.4% of the total costs. In three areas, there is considerable potential for savings. First, more nonphysician staff could be employed in CCCCs; this is already being implemented at LUH as a consequence of this analysis. Second, the cost-efficiency analysis shows potential for optimization in the utilization of the manpower of the personnel deployed. Third, artificial intelligence solutions are becoming increasingly more relevant in the COVID-19 pandemic for diagnosis, public health, clinical decision-making, and therapeutics, and they could possibly replace human-based decisions in the future [33].

In the months considered here, with substantially higher incidence of COVID-19, hospitals needed to implement a noticeably lower reduction in surgery and treatment capacity than in the first pandemic wave (compare Dercks et al [34]). This also results from the improved distribution of patients with COVID-19 and more predictable planning by the CCCC, among others. In view of the expenses for the CCCC, which are partly compensated by the state government of Saxony, an efficient allocation of patients with COVID-19 by the CCCC will result in real cost savings. These savings can be seen not only for the LUH but for all hospitals under the CCCC’s coordination as local overload.

Nonfinancial benefits of the CCCC are particularly evident in two areas. First, unmanageable situations in the ED (and ICU) as well as insufficient human and material resources were prevented at all times. In 11% of the requests, a presentation at the hospital was not necessary and could be anticipated. In addition, it was possible to allocate the patient presentations based on the current capacity of the ED, normal wards, and ICU, as well as the expected necessary medical resources. By giving advance notice prior to admission, necessary preparations could be made to minimize the risk of infection to staff and other patients. Second, the CCCC has a relevant effect in binding the EMS and referring physicians to the CCCC hospital (>90% involvement of the CCCC). We frequently received feedback from referring physicians on how satisfied they were with the fast and competent consultation that was provided (so that we will also consider offering the telemedical consultation in other areas in the future).

### Limitations

Concerning the systematic review, relevant publications may not have been detected due to the search algorithm and screening. As no further relevant publications were found during the reference screening, we consider this limitation to be minor.

The costs of the CCCC are based on real costs (eg, human resources) or general calculation parameters (eg, operating costs/m^2^) of the LUH. In the cost-effectiveness analysis, the workload was calculated using data from a similar but different period. The 2 months that have been taken into consideration in this study correspond to the peak of the pandemic in Saxony to date; therefore, the effectiveness for the total duration of the CCCC could be overestimated. In the use and utility analysis, we cannot directly attribute the requests to patients in the ED due to privacy concerns. We consider the limitations of the economic evaluation mentioned to be minor. A substantial limitation can be seen in that a complete and valid cost-benefit analysis could not be performed since the business year is still ongoing. This should be further investigated in future studies.

### Conclusions

In summary, the establishment and operation of the CCCC has proved worthwhile. Despite the additional costs for the providing hospital, one can assume a significant reduction of financial risks for the hospital itself as well as for the public health system. Potential savings points and future development opportunities could be identified. The most important benefit of the CCCC, however, is that there was no time when hospitals were overrun and no lives had to be triaged as a result.

### Data Availability

The data provided in this study can be obtained in the *Methods* section of this manuscript.

## Abbreviations

CCCC: Central COVID-19 Coordination Center
CHEERS: Consolidated Health Economic Evaluation Reporting Standards
ECMO: extracorporeal membrane oxygenation
ED: emergency department
EMS: emergency medical services
GCS: Glasgow Coma Scale
ICU: intensive care unit
LUH: Leipzig University Hospital
PCR: polymerase chain reaction
PRISMA: Preferred Reporting Items for Systematic Reviews and Meta-Analyses
qSOFA: quick sequential organ failure assessment score
